# Serum melatonin levels in survivor and non-survivor patients with traumatic brain injury

**DOI:** 10.1186/s12883-017-0922-2

**Published:** 2017-07-19

**Authors:** Leonardo Lorente, María M. Martín, Pedro Abreu-González, Antonia Pérez-Cejas, Luis Ramos, Mónica Argueso, Jordi Solé-Violán, Juan J. Cáceres, Alejandro Jiménez, Victor García-Marín

**Affiliations:** 1 Intensive Care Unit, Hospital Universitario de Canarias, Ofra s/n. La Laguna, 38320 Santa Cruz de Tenerife, Spain; 2 Intensive Care Unit, Hospital Universitario Nuestra Señora de Candelaria, Crta del Rosario s/n, 38010 Santa Cruz de Tenerife, Spain; 3 Deparment of Physiology. Faculty of Medicine, University of the La Laguna, Ofra s/n. La Laguna, 38320 Santa Cruz de Tenerife, Spain; 4 Laboratory Deparment, Hospital Universitario de Canarias, Ofra, s/n. La Laguna, 38320 Tenerife, Spain; 5 Intensive Care Unit, Hospital General La Palma, Buenavista de Arriba s/n, 38713 Breña Alta, La Palma, Spain; 6 Intensive Care Unit, Hospital Clínico Universitario de Valencia, Avda. Blasco Ibáñez n°17-19, 46004 Valencia, Spain; 7 Intensive Care Unit, Hospital Universitario Dr. Negrín. CIBERES, Barranco de la Ballena s/n, 35010 Las Palmas de Gran Canaria, Spain; 8 Intensive Care Unit, Hospital Insular, Plaza Dr. Pasteur s/n, 35016 Las Palmas de Gran Canaria, Spain; 9 Research Unit, Hospital Universitario de Canarias, Ofra s/n. La Laguna, 38320 Santa Cruz de Tenerife, Spain; 10 Deparment of Neurosurgery, Hospital Universitario de Canarias, Ofra, s/n. La Laguna, 38320 Santa Cruz de Tenerife, Spain

**Keywords:** Melatonin, Brain trauma, Patients, Mortality, Injury

## Abstract

**Background:**

Circulating levels of melatonin in patients with traumatic brain injury (TBI) have been determined in a little number of studies with small sample size (highest sample size of 37 patients) and only were reported the comparison of serum melatonin levels between TBI patients and healthy controls. As to we know, the possible association between circulating levels of melatonin levels and mortality of patients with TBI have not been explored; thus, the objective of our current study was to determine whether this association actually exists.

**Methods:**

This multicenter study included 118 severe TBI (Glasgow Coma Scale <9) patients. We measured serum levels of melatonin, malondialdehyde (to assess lipid peroxidation) and total antioxidant capacity (TAC) at day 1 of severe TBI. We used mortality at 30 days as endpoint.

**Results:**

We found that non-survivor (*n* = 33) compared to survivor (*n* = 85) TBI patients showed higher circulating levels of melatonin (*p* < 0.001), TAC (*p* < 0.001) and MDA (*p* < 0.001). We found that serum melatonin levels predicted 30-day mortality (Odds ratio = 1.334; 95% confidence interval = 1.094–1.627; *p* = 0.004), after to control for GCS, CT findings and age. We found a correlation between serum levels of melatonin levels and serum levels of TAC (rho = 0.37; *p* < 0.001) and serum levels of MDA (rho = 0.24; *p* = 0.008).

**Conclusions:**

As to we know, our study is the largest series providing circulating melatonin levels in patients with severe TBI. The main findings were that non-survivors had higher serum melatonin levels than survivors, and the association between serum levels of melatonin levels and mortality, peroxidation state and antioxidant state.

## Background

Severe traumatic brain injury (TBI) leads to resources consumption, disabilities, and deaths [1]. TBI causes primary and secondary brain injuries. Primary brain injury is produced due to physical forces at the moment of impact. Secondary brain injury, during following hours or days to TBI, leads to neuroinflammation and brain oxidative damage [2–6].

There has been suggested that the administration of melatonin on TBI could have different beneficial effects, such as antioxidant effects, anti-inflammatory effects, anti-apoptotic effects, a reduction in brain edema [2–6].

Circulating levels of melatonin in patients with traumatic brain injury (TBI) have been determined in a little number of studies with small sample size [7–11] (the highest sample size was of 37 patients [11]). Some studies found lower melatonin levels in salive [7] or serum [8–10] in TBI patients compared to healthy controls; however, one study found higher levels of melatonin in cerebrospinal fluid in TBI patients compared to healthy controls [11]. As to we know, circulating melatonin levels in survivor and non-survivor patients with TBI, the possible association between serum levels of melatonin levels and mortality and peroxidation state in patients with TBI, and the possible prognostic value of serum levels of melatonin in patients with TBI have not been explored; thus, the objectives of our current study were to determine whether those acts actually exists. For those reasons, we determined in our study serum levels of melatonin, malondialdehyde (to assess lipid peroxidation) and total antioxidant capacity (to assess antioxidant state). The clinical interest of this study lies in that if we find these associations, then the determination of serum melatonin levels in clinical practice to estimate the prognosis of the patients could be proposed, and new lines of research in the treatment of these patients could be opened.

## Methods

### Design and subjects

This was a prospective and observational study in 6 spanish Intensive Care Units, and was approved by the Ethic Review Board of all participating hospitals: H. U. de Canarias (La Laguna), H. U. Nuestra Señora de Candelaria (Santa Cruz de Tenerife), H. General de La Palma (La Palma), H. U. de Valencia (Valencia), H. U. Insular (Las Palmas de Gran Canaria), H. U. Dr. Negrín (Las Palmas de Gran Canaria). Legal guardians of patients written the informed consent. The study adheres to the ethical conduct of research involving human subjects by World Medical Association Declaration of Helsinki.

We included 118 severe TBI patients. We classified TBI severity by Glasgow Coma Scale (GCS) [12]. We defined severe TBI as GCS < 9 points.

We excluded patients with Injury Severity Score (ISS) in non-cranial aspects > 9 points [13], inflammatory or malignant disease, age < 18 years, and pregnancy.

Previously, we used the same patient cohort with severe TBI with lower number of patients (*n* = 100) to analyse serum concentrations of malondialdehyde (MDA) [14] and total antioxidant capacity (TAC) [15]. The aims of our current research were to analyse serum concentrations of melatonin in 118 patients with severe TBI, and the association between serum concentrations of melatonin, MDA and TAC in patients with a severe TBI.

### Variables recorded

In each patient were recorded the following variables: temperature, sex, sodium, glycemia (basal determination previously to start nutrition), leukocytes, bilirrubin, creatinine, hemoglobin, lactic acid, ISS, platelets, GCS, international normalized ratio (INR), activated partial thromboplastin time (aPTT), fibrinogen, Acute Physiology and Chronic Health Evaluation II (APACHE II) score [16], age, and brain lesion according to the Marshall computed tomography (CT) classification [17]. Serum samples and clinical variables were recorded approximately at the same moment.

CT lesion according Marshall classification [17] is as follows: Class I (not visible pathology), Class II (cisterns are present and midline shift < 5 mm and there is not lesion > 25 cm^3^), Class III (cisterns are compressed and midline shift < 5 mm and there is not lesion > 25 cm^3^), Class IV (midline shift > 5 mm and there is not lesion > 25 cm^3^), Class V (evacuated lesion) or Class VI (lesion > 25 cm^3^ not surgically evacuated).

### End-point

We used mortality at 30 days as endpoint.

### Blood sample collection

We collected blood samples on day 1 of TBI (within 4 h after TBI) in tubes with separator gel. After 10 min at room temperature, serum was obtained by centrifugation during 15 min at 1000 g, and frozen at −80 °C on each hospital until serum concentration determinations. Afterwards, the samples were transported between different locations in refrigerated boxes with dry ice.

### Determination of serum levels of melatonin, total antioxidant capacity (TAC) and malondialdehyde (MDA)

All serum concentrations were determined at the same moment (to avoid the possible dispersion of results), when recruitment process was finished, and were determined by personnel without access to clinical data.

The determination of total antioxidant capacity (TAC) give more information about patient antioxidant status than determining concentrations of each antioxidant compounds [18]. It is due to that antioxidant compounds establish complex interactions with other antioxidant compounds and do not work alone [19].

Malondialdehyde (MDA) appears during the lipid peroxidation from cellular membrane phospholipids degradation, afterwards is released into extracellular space, and finally appears in the blood [20, 21].

In the Physiology Department at Faculty of Medicine of University of La Laguna (Tenerife, Spain) was performed the determination of serum concentrations of melatonin using a kit from Immuno Biological Laboratories (IBL Hamburg GmbH, Hamburg, Germany) based in ELISA method, which has a detection limit of 0.13 pg/ml, an intra-assay coefficient of variation (CV) of 6.4% and an inter-assay CV of 11.1%.

In the Laboratory Department at Hospital Universitario de Canarias (La Laguna, Tenerife, Spain) was performed the determination of serum concentrations of TAC using a kit from Cayman Chemical Corporation (Ann Arbor, USA) based in the ability of the sample to inhibit the oxidation of 2,2′-azino-di-[3-ethylbenzthiazoline sulphonate] (ABTS) by metmyoglobin, which has a detection limit of 0.04 mmol/L, an intra-assay CV of 3.4% and an inter-assay CV of 3.0%.

In the Physiology Department at Faculty of Medicine of University of La Laguna (Tenerife, Spain) was performed the determination of serum concentrations MDA using a thiobarbituric acid-reactive substance (TBARS) assay according to Kikugawa et al. [22], which has a detection limit of 0.079 nmol/mL, an intra-assay CV of 1.82% and an inter-assay CV of 4.01%.

### Statistical methods

Medians and interquartile ranges were used to report continuous variables, and frequencies and percentages to report categorical variables. Wilcoxon-Mann-Whitney test was used to compare continuous variables between groups, and chi-square test to compare categorical variables.

We used logistic regression analysis to determine the association with 30-day mortality. We carried out two logistic regression models with four variables because 33 patients died. We included in logistic regression analysis the statistically significant variables in the bivariate analysis. We included serum melatonin levels, GCS, CT classification, and age in the first model. We included serum melatonin levels, APACHE-II score, CT classification, and sex in the second model. We recoded the CT classification variable previously to include it in the logistic regression analysis. We found the following mortality rates according CT classification: 5/29 (12.5%) in patients with class 2, 6/20 (30.0%) with class 3, 9/21 (42.9%) with class 4, 5/35 (14.3%) with class 5 and 8/13 (61.5%) with class 6. Then we recoded the CT classification variable as low risk of death (CT class 2 or 5) and high risk of death (CT class 3, 4 or 6). The group of patients with CT with low risk of death (CT class 2 or 5) had a mortality rate of 10/64 (15.6%); and the group of patients with CT with high risk of death (CT class 3, 4 or 6) had a mortality rate of 23/54 (42.6%). We calculate Odds Ratio and 95% confidence intervals to measure the association of variables with mortality.

Receiver operator characteristic (ROC) curve was constructed with serum levels of melatonin as prognostic variable and 30-day survival as classification variable. To select cut-off prognostic value of serum melatonin level (3.53 pg/mL), Youden J index was used**.** Kaplan-Meier analysis was constructed with survival at 30 days and with cut-off serum melatonin levels (> or <3.53 pg/mL), and both curves were compared using log-rank test.

Correlation between continuous variables was analysed using coefficient of Spearman. We considered *p*-values < 0.05 as statistically significant. NCSS 2000 (Kaysville, Utah), SPSS 17.0 (SPSS Inc., Chicago, IL, USA), and LogXact 4.1, (Cytel Co., Cambridge, MA) were used to carry out statistical analyses.

## Results

Biochemical and clinical characteristics of survivor (*n* = 85) and non-survivor (*n* = 33) TBI patients are showed in Table 1. Non-survivor TBI patients compared to survivor had higher serum levels of melatonin (*p* < 0.001), TAC (*p* < 0.001), and MDA (*p* < 0.001). In addition, non-survivor TBI patients compared to survivor patients had higher APACHE-II score, female rate and age, and lower GCS than survivors. Besides, non-survivor and survivor TBI patients had differences in CT classification.

**Table 1:** Clinical and biochemical characteristics of survivor and non-survivor patients

	Non-survivors (*n* = 33)	Survivors (*n* = 85)	*P value*
Gender female – n (%)	13 (39.4)	15 (17.6)	0.02
Age (years) - median (p 25–75)	66 (55–75)	46 (28–62)	<0.001
Temperature (°C) - median (p 25–75)	36.0 (35.0–37.0)	37.0 (36.0–37.3)	0.12
Sodium (mEq/L)- median (p 25–75)	142 (138–148)	140 (138–143)	0.19
Glycemia (g/dL) - median (p 25–75)	160 (134–189)	139 (122–167)	0.08
Leukocytes-median*10^3^/mm^3^ (p 25–75)	16.3 (9.8–22.7)	14.5 (10.3–19.0)	0.46
PaO2 (mmHg) - median (p 25–75)	133 (98–180)	148 (110–203)	0.34
PaO2/FI0_2_ ratio - median (p 25–75)	274 (173–393)	336 (240–400)	0.11
Bilirubin (mg/dl) - median (p 25–75)	0.70 (0.58–0.95)	0.50 (0.40–0.80)	0.045
Creatinine (mg/dl) - median (p 25–75)	0.80 (0.70–1.10)	0.80 (0.63–1.00)	0.44
Hemoglobin (g/dL) - median (p 25–75)	11.9 (9.8–13.1)	11.4 (10.2–13.0)	0.87
GCS score - median (p 25–75)	3 (3–6)	7 (5–8)	<0.001
Lactic acid (mmol/L)-median (p 25–75)	2.40 (1.30–4.60)	1.70 (1.10–2.50)	0.06
Platelets - median*10^3^/mm^3^ (p 25–75)	180 (125–237)	184 (134–244)	0.52
INR - median (p 25–75)	1.12 (1.03–1.40)	1.11 (1.00–1.21)	0.29
aPTT (seconds) - median (p 25–75)	29 (25–36)	28 (25–31)	0.31
Fibrinogen (mg/dl) - median (p 25–75)	361 (269–520)	366 (283–448)	0.99
APACHE-II score - median (p 25–75)	25 (23–28)	18 (14–22)	<0.001
ISS - median (ppe 25–75)	25 (25–25)	25 (25–29)	0.43
ICP (mmHg) - median (p 25–75)	25 (13–34)	15 (14–20)	0.28
CPP (mmHg) - median (p 25–75)	61 (54–69)	68 (57–70)	0.48
CT classification - n (%)			0.006
Type 1	0	0	
Type 2	5 (15.2)	24 (28.2)	
Type 3	6 (18.2)	14 (16.5)	
Type 4	9 (27.3)	12 (14.1)	
Type 5	5 (15.2)	30 (35.3)	
Type 6	8 (24.2)	5 (5.9)	
CT with high risk of death (types 3,4,6)- n (%)	23 (69.7)	31 (36.5)	0.002
Melatonin (pg/mL) - median (p 25–75)	6.74 (3.78–7.34)	2.49 (2.13–3.24)	<0.001
TAC (mmol/mL) - median (p 25–75)	5.09 (2.45–9.63)	2.36 (1.82–2.94)	<0.001
MDA (nmol/mL) - median (p 25–75)	2.01 (1.35–4.24)	1.36 (1.05–1.79)	<0.001

*P 25–75* percentile 25th - 75th, *PaO*_*2*_ pressure of arterial oxygen/fraction inspired oxygen, *FIO*_*2*_ pressure of arterial oxygen/fraction inspired oxygen, *GCS* Glasgow Coma Scale, *ISS* Injury Severity Score, *INR* international normalized ratio, *aPTT* activated partial thromboplastin time, *APACHE II* Acute Physiology and Chronic Health Evaluation, *ICP* intracranial pressure, *CPP* cerebral perfusion pressure, *CT* computer tomography, *TAC* total antioxidant capacity, *MDA* Malondialdehyde

Logistic regression analyses are showed in Table 2. Serum melatonin levels are associated with 30-day mortality (OR = 1.334; 95% CI = 1.094–1.627; *p* = 0.004) after to control for age, GCS and CT lesions. Besides, serum melatonin levels are associated with 30-day mortality (OR = 1.364; 95% CI = 1.108–1.678; *p* = 0.003) after to control for sex, APACHE-II and CT lesions.

**Table 2:** Multiple binomial logistic regression analysis to predict 30-day mortality

Variable	Odds Ratio	95% Confidence Interval	*P*
First Model			
Serum melatonin levels (pg/mL)	1.334	1.094–1.627	0.004
GCS score (points)	0.574	0.426–0.773	<0.001
Age (years)	1.047	1.014–1.082	0.006
Computer tomography classification (reference category: low risk of death)	4.526	1.375–14.895	0.013
Second Model			
Serum melatonin levels (pg/mL)	1.364	1.108–1.678	0.003
APACHE-II score (points)	1.315	1.148–1.505	<0.001
Sex (reference category: female)	0.477	0.125–1.823	0.280
Computer tomography classification (reference category: low risk of death)	4.360	1.246–15.253	0.021

*GCS* Glasgow Coma Scale, *APACHE II* Acute Physiology and Chronic Health Evaluation

**Fig. 1:**
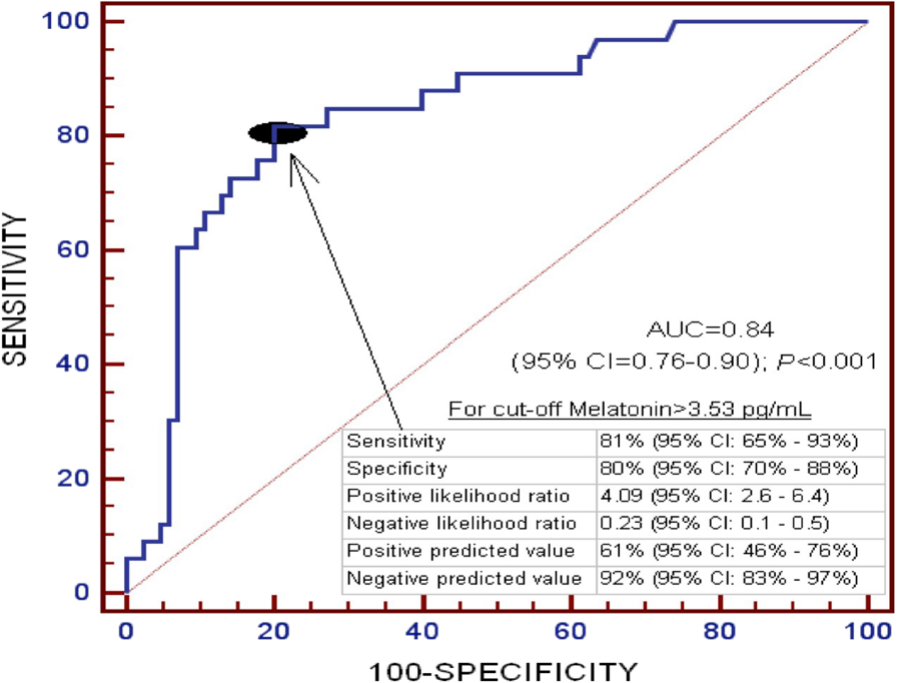
Receiver operation characteristic analysis using serum melatonin levels as predictor of mortality at 30 days

Area under the curve (AUC) to predict 30-day mortality for serum melatonin levels was of 0.84 (95% CI = 0.76–0.90; *p* < 0.001) (Fig. 1). We have not found differences in AUC for GCS (0.77; 95% CI = 0.68–0.84) and serum melatonin levels (*p* = 0.19).

**Fig. 2:**
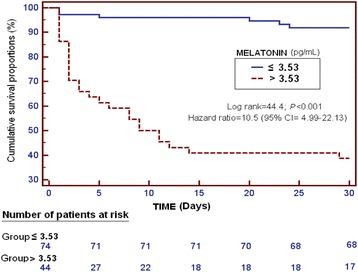
Survival curve at 30 days using 3.53 pg/mL of serum melatonin levels as cut-off

Kaplan-Meier analysis showed a higher mortality at 30 days in patients with serum melatonin levels > 3.53 pg/mL (Hazard ratio = 10.5; 95% CI = 4.99–22.13; *p* < 0.001) (Fig. 2).

We found a positive correlation of serum melatonin levels with serum TAC levels (rho = 0.37; *p* < 0.001), and with serum MDA levels (rho = 0.24; *p* = 0.008).

## Discussion

As we know, this study is the series of higher sample size providing serum levels of melatonin levels in patients with TBI. The new findings were that non-survivor compared to survivor patients had higher serum melatonin levels, that an association between serum melatonin levels, peroxidation state, antioxidant state, and mortality exists, and that serum melatonin levels could be used as biomarker for mortality prediction in patients with TBI.

Previously, circulating levels of melatonin in patients with TBI have been determined in a little number of studies [7–11], and was reported only the comparison of serum melatonin levels between TBI patients and healthy controls. Thus, our study is the first providing serum melatonin levels in survivor and non-survivor patients with TBI, and higher levels in non-survivors compared to survivors. Besides, to our knowledge, our study (including 118 TBI patients) is the study with largest sample size reporting serum melatonin levels in patients with TBI.

In the Seifman et al. study [11] was found a weak positive correlation of serum levels of melatonin and 6-month Glasgow outcome score extended (GOSE) in 39 severe TBI patients (*r* = 0.189; *p* = 0.004), and higher serum melatonin levels may indicate a better outcome. Our findings are in contrast with those of Seifman et al. Posibles explanations for those different findings could be the outcome chosen (mortality in our study, and GOSE in the study of Seifman et al.), the moment to assess the outcome (30 days in our study, and 6 months in the work by Seifman et al.), and the sample size (118 patients in our study, and 39 patients in the research of Seifman et al.). We chosen mortality at 30 days as outcome due to that in previous studies by us and by other researchers was used this outcome.

In addition, we found that an association of serum levels of melatonin with mortality exists in patients with TBI for the first time. Besides, to our knowledge, our study is the first suggested that serum melatonin levels could be used as biomarker for mortality prediction in patients with TBI. Also, as to we know, the association of serum levels of melatonin, TAC and MDA found in our study has been reported for the first time. We found, as previously were described, higher serum levels of MDA [14, 23] and TAC [15, 24] in non-survivor compared to survivors.

In the Seifman et al. study were found higher levels of melatonin and isoprostane (to assess oxidative stress) in cerebrospinal fluid in TBI patients compared to healthy controls, and a positive association of cerebrospinal fluid levels of melatonin with isoprostane [11]. However, in the study by Seifman et al. were not found differences in serum levels of melatonin in TBI patients compared to healthy controls, and they found a negative association of serum levels of melatonin with isoprostane. These authors believed that the increase of melatonin levels in CSF after TBI likely represents a response to oxidative stress. Besides, they believed that that negative correlation of serum levels of melatonin with isoprostane that they found could represent that patients with higher oxidative stress may have higher melatonin consumption and antioxidant activity.

We think that non-survivor compared to survivor TBI patients have higher ROS production, which leads to a higher oxidant state (assessed by increased serum levels of MDA); and that increased serum levels of TAC and melatonin are an attempt to maintain the balance between oxidant and antioxidant state due to the high production of oxidant products. However, in non-survivors those increase of serum levels of TAC and melatonin are not enough for the compensation of high production of oxidants species and then they present higher peroxidation of proteins, lipids, carbohydrates and nucleic acids, contributing to cellular dysfunction, vasogenic edema [2–6].

The administration of melatonin could be suggested for the treatment of patients with severe TBI according to animal model results [25–33]. The administration of melatonin has been associated with antioxidant effects [25–31], anti-inflammatory effects [28, 33], a reduction in brain edema [29, 30, 32], and anti-apoptotic effects [31]. We think that the melatonin administration could help to compensate the increased oxidant products production in patients with higher oxidant state and higher risk of death. In addition, we think that the melatonin administration could contribute to reduce cellular dysfunction and vasogenic edema, and finally reduce the risk of death.

Our study presents several limitations. First, serum melatonin levels in non-surviving and surviving during follow-up were not described. Second, the determination of other compounds of oxidant and antioxidant states would have been interesting. Third, we did not analyse concentrations of melatonin in cerebrospinal fluid; however, we proposed a low invasiveness protocol and this was the motive to determine melatonin levels in serum. Fourth, due to that the objective of the study was recollect blood samples early after TBI (within 4 h after TBI) then the moment of day to obtain blood samples was not the same for all patients, and the melatonin circadian rhythm is known. Fifth, serum melatonin concentations in control subjects were not analysed; however, our objective study was to analyse whether there is an association of serum melatonin levels with 30-day mortality, and not to determine whether severe TBI modify serum melatonin levels. However, as circulating melatonin levels could be differents according to laboratory kits and moment of day, then the determinations in control subjects could have been interesting. Finally, we think that our study could open the perspective for an interventional trial in patients with TBI.

## Conclusions

As to we know, our study is the largest series providing circulating melatonin levels in patients with severe TBI. The main findings were that non-survivors had higher serum melatonin levels than survivors, and the association between serum levels of melatonin levels and mortality, peroxidation state and antioxidant state.

## Abbreviations

APACHE: Acute physiology and chronic health evaluation
APTT: Activated partial thromboplastin time
CPP: Cerebral perfusion pressure
CT: Computed tomography
FIO_2_: Fraction inspired of oxygen
GCS: Glasgow coma scale
ICP: Intracranial pressure
ICU: Intensive care unit
INR: International normalized ratio
ISS: Injury severity score
MDA: Malondialdehyde
PaO_2_: Pressure of arterial oxygen
SOFA: Sepsis-related organ failure assessment score
TAC: Total antioxidant capacity
TBI: Trauma brain injury
